# Serum lipid levels in pregnancies complicated by preeclampsia

**DOI:** 10.1590/S1516-31802011000200004

**Published:** 2011-03-03

**Authors:** Valmir Jose de Lima, Claudia Roberta de Andrade, Gustavo Enrico Ruschi, Nelson Sass

**Affiliations:** I MD, MSc. Gynecologist and Obstetrician, Obstetrics Department, Universidade Federal de São Paulo (Unifesp), São Paulo, São Paulo, Brazil.; II PhD. Professor, Universidade Federal do Ceará (UFC), Fortaleza, Ceará, Brazil.; III MD. Attending Physician, Santa Casa de Vitória Medical School, Vitória, Espírito Santo, Brazil.; IV MD,PhD. Associate Professor, Obstetrics Department, Universidade Federal de São Paulo (Unifesp), São Paulo, São Paulo, Brazil.

**Keywords:** Lipids, Hypertension, pregnancy-induced, Pre-eclampsia, Cholesterol, Hyperlipidemias, Lipídeos, Hipertensão induzida pela gravidez, Pré-eclâmpsia, Colesterol, Hiperlipidemias

## Abstract

**CONTEXT AND OBJECTIVE:**

Pre-eclampsia is a disorder that occurs only during pregnancy. Postpartum changes relating to lipid metabolism may contribute towards the endothelial lesions observed in preeclampsia. Thus, the aim of the present study was to evaluate the lipid profile among patients who present preeclampsia and correlate these parameters with 24-hour proteinuria.

**DESIGN AND SETTING:**

Cross-sectional analytical study including 77 pregnant patients seen at Hospital Dório Silva.

**METHODS:**

This study involved 42 women with preeclampsia and 35 healthy pregnant women in the third trimester of pregnancy as controls. Blood samples were obtained from all the patients, and the serum levels of triglycerides, total cholesterol, low-density lipoproteins (LDL), high-density lipoproteins (HDL) and very low density lipoproteins (VLDL) were determined. Cases and controls were matched for maternal age, gestational week and body mass index.

**RESULTS:**

The VLDL and triglyceride values from the women with preeclampsia were significantly higher than those of the healthy women. There was a positive correlation between increased proteinuria and higher VLDL and triglyceride levels in patients with preeclampsia.

**CONCLUSION:**

Among the patients with preeclampsia, higher VLDL and triglyceride levels were positively correlated with proteinuria. These observations indicate that the pregnant women who presented elevated lipid levels were more susceptible to cardiovascular disorders and, consequently, pre-eclampsia.

## Abstract (pt)

**CONTEXTO E OBJETIVO:**

A pré-eclâmpsia é um distúrbio que ocorre apenas durante a gravidez e as alterações pós-parto relacionadas ao metabolismo lipídico podem contribuir para a lesão endotelial observada nessa afecção. Assim, o objetivo do presente estudo foi avaliar o perfil lipídico em pacientes que apresentaram pré-eclâmpsia e correlacionar estes parâmetros com a proteinúria de 24 horas.

**TIPO DE ESTUDO E LOCAL:**

Estudo transversal analítico, incluindo 77 pacientes grávidas atendidas no Hospital Dório Silva.

**MÉTODOS:**

Este estudo envolveu 42 mulheres com pré-eclâmpsia e 35 gestantes saudáveis no terceiro trimestre de gravidez como controle. Amostras de sangue foram obtidas de todas as pacientes e os níveis séricos de triglicérides, colesterol total, lipoproteínas de baixa densidade (LDL), lipoproteínas de alta densidade (HDL) e lipoproteínas de muito baixa densidade (VLDL) foram determinados. Casos e controles foram pareados por idade materna, semana gestacional e índice de massa corporal.

**RESULTADOS:**

Os valores de VLDL e triglicérides, obtidos de mulheres com pré-eclâmpsia foram significativamente maiores quando comparados com mulheres saudáveis. Houve uma correlação positiva entre o aumento da proteinúria e concentrações elevadas de VLDL e triglicérides, em pacientes com pré-eclâmpsia.

**CONCLUSÃO:**

Nas pacientes com pré-eclâmpsia foram observadas as maiores concentrações de VLDL e os níveis de triglicérides estão correlacionados positivamente com proteinúria. Estas observações indicam que as mulheres grávidas que apresentam níveis elevados de lipídios, são mais suscetíveis a doenças cardiovasculares e, consequentemente, a pré-eclâmpsia.

## INTRODUCTION

Pre-eclampsia is a disorder that occurs only during pregnancy and the postpartum period. It affects both the mother and the unborn baby and occurs in approximately 5% of all pregnancies, being an important cause of maternal morbidity and mortality.^1,2^ Despite extensive investigations, important pathophysiological aspects of this disease remain unknown, thus delaying the development of preventive and therapeutic strategies.

This disorder is mediated by placental products that reach the maternal circulation and trigger endothelial dysfunction, thereby evoking cardiovascular diseases, such as vasospasm, increased endothelial permeability and activation of thrombogenic mechanisms, and leading to the early events of atherosclerosis.^3^ Susceptibility to preeclampsia is also modulated by maternal factors, and women who present chronic hypertension, diabetes or hyperlipidemia are more likely to exhibit intense vascular reactivity, which evokes important disorders of physiological conditions.

Women with preeclampsia present arterial lesions at the uteroplacental implantation site. These morphological lesions are usually observed in cases of acute atherosclerosis, and are characterized by areas with fibrinoid necrosis surrounded by lipid-laden macrophages.^4^

These microscopic lesions are similar to atherosclerosis found outside pregnancy. Lipid deposits are also seen in the glomeruli of preeclamptic patients, a finding known as glomerular endotheliosis. Glomerular lesions are associated with proteinuria, a predictive indicator and marker of disease severity.^3^ It has also been suggested that low-density lipoproteins (LDL)^4^ and triglycerides^5-7^ may be involved in this renal damage.

Furthermore, changes to lipid metabolism may contribute towards the endothelial lesions observed in preeclampsia.^8^ The severity of both hypertension and proteinuria seems to reflect the degree of endothelial damage.^9,10^The possible correlation between the altered lipid profile and the severity of renal lesions, as reflected by proteinuria, may contribute towards clarify the complex pathophysiology of preeclampsia.

## OBJECTIVE

The aim of this study was to analyze the lipid profile among patients with preeclampsia, and compare it with that observed among healthy pregnant women. A second objective was to correlate these lipid profile findings among preeclamptic patients with the severity of proteinuria.

## PATIENTS AND METHODS

### Study design and size

We conducted a cross-sectional study that included 77 patients: 42 women with preeclampsia and 35 healthy women. The patients were divided into two groups: pregnant women with preeclampsia (cases) and normal pregnant women (controls). The cases and controls were matched according to maternal age, gestational age, race and body mass index (BMI), in accordance with the selection criteria previously established. The study group and controls were selected from among the patients seen at Hospital Dória Silva, Vitória, Espírito Santo, Brazil, between June 2005 and January 2008. The study was approved by the Ethics Committees of Hospital São Paulo, Universidade Federal de São Paulo — Escola Paulista de Medicina (Unifesp-EPM) number 0188/05 and of Hospital Dória Silva, and written informed consent was obtained from all patients.

The sample size was calculated to compare two groups (cases and controls) with a 5% prevalence of preeclampsia, confidence level of 95% and a power of 80%. In this case, it was expected that the maximum difference in proteinuria in the test group would be 30%, compared with the unexposed group. Thus, the sample size would be a minimum of 33 women in each group.

### Measurements and lab tests

All the women seen at the hospital in the third trimester of singleton pregnancies were considered eligible for inclusion in the study. Gestational age was based on menstrual date and confirmed through ultrasound. Women with diabetes, chronic hypertension, autoimmune diseases or renal diseases were excluded. Preeclampsia was diagnosed in accordance with the criteria proposed by the National High Blood Pressure Education Program,^10^ i.e. blood pressure ≥ 140/90 mmHg and proteinuria ≥ 300 mg in 24 hour urine samples.

Blood samples were collected from all participants after a 12-hour fast using 5 ml tubes containing ethylenediaminetetraacetic acid (EDTA). The samples were immediately centrifuged and processed using a lab test diagnostic kit. The serum levels of triglycerides, total cholesterol, LDL, HDL and VLDL were interpreted in accordance with the recommendations of the National Cholesterol Education Program (Programa Nacional de Educação para o Colesterol, PNEC).^11^ Preeclamptic patients were then asked to collect urine for 24 hours for proteinuria quantification. This was done by means of photometric readings after addition of sulfosalicylic or trichloroacetic acid.

### Statistical analysis

The mean serum lipid concentrations of the cases and controls were compared using Student's t test. The mean level of each lipid was correlated with the 24 hour proteinuria concentration using Pearson's coefficient test. Significance was set at P < 0.05. We also used the Student t test to compare the means of the groups for arterial pressure, proteinuria, total cholesterol, high-density lipoproteins (HDL), LDL, very low density lipoproteins (VLDL) and triglycerides, taking P < 0.05. Correlations between proteinuria and cholesterol were made using Pearson's correlation coefficient, considering only the group with preeclampsia. The analyses were performed using the EpiInfo software, version 6.

## RESULTS

During the study period, 42 preeclamptic and 35 healthy pregnant women fulfilling the inclusion criteria were invited to participate in the study. The participants' characteristics are presented in Table 1 and the data demonstrate that there were no significant differences in the women's ages, extent of pregnancy, body mass index (BMI) or race.

**Table 1.** Demographic and clinical characteristics of participants

Characteristic	Preeclampsia n = 42	Healthy controls n = 35	P
Race/ethnicity			
White	19 (45.2)	12 (34.3)	0.6*
Mixed	20 (47.6)	19 (54.3)	
Black	3 (7.1)	4 (11.4)	
Age	23.5 ± 4.7	23.3 ± 4.8	0.9†
Body mass index	27.6 ± 3.3	27,9 ± 3.6	0.7†
Gestational age	36.0 ± 2.7	36.7 ± 1.7	0.2†

* Chi-square

† Student t test. Values expressed in numbers and percentages or mean and standard deviation.

Furthermore, there were no significant differences in the total serum cholesterol, LDL and HDL levels between the preeclampsia cases and the healthy women. The preeclamptic patients had significantly higher serum levels of triglycerides and VLDL, compared with the healthy controls (Table 2). The triglyceride and VLDL levels were positively and significantly correlated with the severity of proteinuria (Table 2).

**Table 2.** Correlation between 24-hour proteinuria and lipid profile among 72 pregnant women

Proteinuria (mg/dl over 24 hours)					
	Absent	0.3 – 0.9	1 – 2	> 2	P
Total cholesterol	229.0 (53)	225.0 (42)	231.0 (69)	273.0 (65)	0.06
LDL	133.0 (46.7)	121.8 (34.9)	123.0 (42.6)	150.2 (50.4)	0.30
VLDL	43.3 (11.9)	48.3 (18.5)	59.7 (25.7)	67.8 (17.2)	< 0.0001
HDL	53.0 (14)	55.0 (14)	49.0 (12)	56.0 (11)	0.60
Triglycerides	216 (59)	241 (93)	298 (128)	339 (86)	< 0.0001

Lipid values expressed in mg/dl, mean (and standard deviation). LDL = low-density lipoproteins; VLDL = very low density lipoproteins; HDL = high-density lipoproteins.

## DISCUSSION

Hypertensive disorders during pregnancies, named preeclampsia, are a pregnancy-specific disorder that affects 3–5%^12,13^ of pregnant women worldwide. Preeclampsia is one of the most frequently encountered medical complications of pregnancy. Classically, the condition presents with new-onset hypertension and proteinuria after 20 weeks of gestation.^14^ In developing countries where access to healthcare is limited, preeclampsia is a leading cause of maternal mortality, causing an estimated 60,000 maternal deaths worldwide per year.^12^ Furthermore, preeclampsia is the third biggest cause of maternal mortality in the United States and accounts for 20% of maternal deaths.^15^

In Brazil, hypertension during pregnancy is the main cause of maternal death. Recent studies have pointed out that these hypertensive disorders are responsible for approximately 25% of deaths.^16^ Although manageable and preventable, these hypertensive disorders remain the leading cause of maternal death and a serious health problem that still affects a significant number of women.^17^ It is possible to infer that the determinants of maternal mortality due to hypertension during pregnancy are related to inadequate care and difficulties in accessing specialized reference services for high-risk pregnancy during the prenatal period, at childbirth and during the postpartum period, which would be able to provide better qualified and more efficient care.

The preeclamptic patients in our study presented significantly higher concentrations of triglycerides and VLDL than shown by the healthy women. In a review of 22 studies, Ray et al.^18^ reported that women with elevated triglycerides had twice the risk of preeclampsia, and the four studies that adjusted for confounders (age, BMI and parity) indicated that the risk was four times higher, compared with women with normal triglycerides. It was also suggested that triglyceride assessment between 28 and 32 weeks could be predictive of preeclampsia.^19^ Several other investigators have reported that hypertriglyceridemia could be involved in the pathogenesis of hypertensive disorders during pregnancy.^19-23^ In the present study, we also found a significant and positive association between proteinuria and both triglyceride and VLDL levels. These findings suggest that these lipids may be involved in the endothelial damage observed in our preeclampsia patients.

Although it is still unclear whether hypertriglyceridemia becomes a risk factor for preeclampsia or whether there is any causal association between them, high triglyceride levels seem to increase the risk of placental vascular disorders,^19^ which trigger endothelial dysfunction,^24^ atherosclerosis and thrombosis.^20^ The development of atherosclerosis in the placental spiral arteries of preeclamptic women indicates that elevated levels of triglycerides are involved in this disorder.^25^

The fact that the patients with preeclampsia presented dyslipidemia, characterized by high levels of triglycerides and VLDL, indicates that there are common interfaces between preeclampsia and the endothelial lesions that occur in atherosclerosis. Our results allow us to hypothesize that these lesions may evoke adverse cardiovascular events later on during the adulthood of these women. In a systematic review of the literature, Bellamy et al.^26^ reported that women with a history of preeclampsia presented increased risk of cardiovascular disease (risk relative, RR = 3.7), hypertension (RR = 2.16), ischemic heart attack (RR = 1.81), venous thromboembolism (RR = 1.79) and death (RR = 1.49). These findings confirm the possible association between hypertension during pregnancy and future cardiovascular disease.

In order to implement preventive healthcare protocols, it is important to identify patients who are at risk of developing cardiovascular diseases. Better understanding of lipid metabolism abnormalities and of how these changes interact with the endothelial dysfunction of preeclampsia is crucial from a public health perspective.

## CONCLUSIONS

The findings reported in the present study corroborate the growing numbers of studies showing that women with preeclampsia present lipid profile abnormalities and that these lipids become an increased risk factor for cardiovascular complications. Therefore, these women should receive adequate counseling to urge them to adopt healthier habits and lifestyles and to seek periodic checkups, in order to detect cardiovascular disease in its early stages, before irreparable damage or even death ensues.
